# Irisin Ameliorates Intervertebral Disc Degeneration by Activating LATS/YAP/CTGF Signaling

**DOI:** 10.1155/2022/9684062

**Published:** 2022-07-20

**Authors:** Taiqiu Chen, Youxi Lin, Zizhao Wu, Huihong Shi, Wenjun Hu, Shaoguang Li, Yichen Que, Jincheng Qiu, Pengfei Li, Xianjian Qiu, Tongzhou Liang, Xudong Wang, Bo Gao, Hang Zhou, Zhihuai Deng, Yanbo Chen, Yuanxin Zhu, Yan Peng, Anjing Liang, Wenjie Gao, Dongsheng Huang

**Affiliations:** ^1^Department of Orthopedics, Sun Yat-sen Memorial Hospital of Sun Yat-sen University, Guangzhou, Guangdong, China; ^2^Department of Orthopedics, The Third Affiliated Hospital of Sun Yat-sen University, Guangzhou, Guangdong, China; ^3^Musculoskeletal Research Laboratory, Department of Orthopaedics & Traumatology, Faculty of Medicine, The Chinese University of Hong Kong, Hong Kong, China; ^4^Department of Orthopedics, The First Affiliated Hospital of Sun Yat-sen University, Guangzhou, Guangdong, China

## Abstract

Unbalanced metabolism of an extracellular matrix (ECM) in nucleus pulposus cells (NPCs) is widely acknowledged as the primary cause of intervertebral disc degeneration (IDD). Irisin, a novel myokine, is cleaved from fibronectin type III domain-containing 5 (FNDC5) and has recently been proven to regulate the metabolism of ECM. However, little is known about its potential on NPCs and the development of IDD. Therefore, this study sought to examine the protective effects and molecular mechanism of irisin on IDD *in vivo* and *in vitro*. Decreased expression levels of FNDC5 and anabolism markers (COL2A1 and ACAN) but increased levels of catabolism markers (ADAMTS4) were found in degenerative nucleus pulposus (NP) tissues. In a punctured-induced rat IDD model, irisin treatment was found to significantly slow the development of IDD, and in TNF-*α*-stimulated NPCs, irisin treatment partly reversed the disorder of ECM metabolism. In mechanism, RNA-seq results suggested that irisin treatment affected the Hippo signaling pathway. Further studies revealed that with irisin treatment, the phosphorylation levels of key factors (LATS and YAP) were downregulated, while the expression level of CTGF was upregulated. Moreover, CTGF knockdown partially eliminated the protective effects of irisin on the metabolism of ECM in NPCs, including inhibiting the anabolism and promoting the catabolism. Taken together, this study demonstrated that the expression levels of *FNDC5* were decreased in degenerative NP tissues, while irisin treatment promoted the anabolism, inhibited the catabolism of the ECM in NPCs, and delayed the progression of IDD via LATS/YAP/CTGF signaling. These results shed light on the protective actions of irisin on NPCs, leading to the development of a novel therapeutic target for treating IDD.

## 1. Introduction

Intervertebral disc degeneration (IDD), regarded as an integral part of the aging process [1, 2], is characterized by unbalanced metabolism of extracellular matrix (ECM), tissue dehydration, cracks in the annulus fibrosus, and destruction of endplate cartilage [3–5]. Although the pathogenesis of IDD is complex and diverse, impaired ECM metabolism is widely considered to be the core pathological change, including downregulated levels of anabolism markers and upregulated levels of catabolism markers [6, 7]. As degeneration proceeds, more inflammatory mediators are secreted by nucleus pulposus cells (NPCs), causing the secretion of matrix-degrading enzymes and aggravating the unbalanced metabolism of ECM, including the upregulation of anabolism markers and downregulation of catabolism markers, which finally accelerate the progression of IDD [8–10].

TNF-*α* is one of the IDD-related inflammatory cytokines, which induces the expression levels of matrix-degrading enzymes and other inflammatory factors in NPCs and thus exacerbates the inflammatory response and accelerates the progression of IDD [11–13]. In addition, as reported, intradiscal injection with the TNF-*α* reagent was considered to be a practicable way to construct a disc degeneration animal model [14]. Moreover, it had been reported that TNF-*α* was involved in the nerve irritation and ingrowth, inducing disc degeneration and increasing the painful behavior in an animal model [15, 16]. Collectively, searching the effective way to alleviate the inflammatory response and unbalanced ECM metabolism induced by TNF-*α* in NPCs appears vital in the treatment of IDD.

Irisin is a soluble polypeptide fragment with 112 amino acids, formed by the cleavage of type III fibronectin component including protein 5 (FNDC5) [17]. Expanding evidence has revealed that irisin is involved in various pathophysiological processes including musculoskeletal metabolism, lipid and glucose metabolism, neural regulation, inflammation, and degenerative diseases [17–20]. Likewise, accumulating studies have shown that irisin plays an essential role against the progression of skeletal degenerative diseases such as osteoporosis and osteoarthritis [21–24]. Besides, it has been reported that irisin is a powerful regulator of the metabolism of ECM, including upregulating the levels of COL2A1 and ACAN and downregulating the productions of matrix-degrading enzymes in chondrocytes [25, 26]. As yet, however, much remains unknown about irisin's effects on the regulation of ECM metabolism in NPCs in the progression of IDD.

The Hippo signaling pathway has been regarded as a conserved transduction pathway with an essential role in regulating cell cycle and differentiation, maintaining the stability of the internal environment and tissue regeneration [27–29]. Recent studies have shown that Hippo signaling may be involved in the development of IDD. As reported, the key effector, the yes-associated protein (YAP), was downregulated, while the phosphorylated YAP protein was upregulated in degenerative NP tissues [30]. Recently, it has been reported that YAP mediates the unbalanced ECM metabolism in NPCs and is found to be regulative in the progression of IDD [31, 32]. As the target gene of YAP, the connective tissue growth factor (CTGF), also known as CCN2, is a secreted peptide consisting of 349 amino acids, composed of N-terminal signal peptide, intermediate protein-binding domain, and C-terminal-binding domain [33]. CTGF has been demonstrated to be involved in many biological processes such as cell proliferation, differentiation, adhesion, and angiogenesis [33–35]. More importantly, CTGF has reportedly shown protective effects on the metabolism of ECM in NPCs, including upregulating the levels of synthesis markers and inhibiting the levels of matrix-degrading enzymes [36–38]. These studies revealed that the LATS/YAP/CTGF axis played a vital role in the progression of IDD, and as such, further studies are warranted.

Collectively, this study was aimed at exploring whether irisin affects ECM metabolism in NPCs and the development of IDD. Herein, it was reported that irisin reversed the disordered metabolism of ECM and ameliorated the progression of IDD via LATS/YAP/CTGF signaling pathways. These results provided additional evidence for the potential use of irisin as a repair reagent on IDD in the future.

## 2. Materials and Methods

### 2.1. Antibodies and Reagents

Antibodies against collagen type II (COL2A1), a disintegrin-like and metalloprotease with thrombospondin type-1 motif 4 (ADAMTS4), tumor necrosis factor-alpha (TNF-*α*), and a disintegrin-like and metalloprotease with thrombospondin type-1 motif 5 (ADAMTS5) were purchased from Abcam Inc. Antibodies against matrix metalloproteinase 9 (MMP9), matrix metalloproteinase 13 (MMP13), connective tissue growth factor (CTGF), and Hippo signaling relative proteins (LATS1, LATS2, YAP, and phosphor-YAP) and goat anti-rabbit and anti-mouse IgG secondary antibodies were purchased from Cell Signaling Technology Inc. Antibodies against GAPDH and *β*-tubulin were purchased from Proteintech Group Inc. Antibody against fibronectin type III domain-containing protein 5 (FNDC5) was purchased from Bioss Inc. An irisin reagent was purchased from Novoprotein, and a verteporfin reagent was purchased from MedChemExpress, while a human TNF-alpha reagent was from R&D Systems. The catalog numbers and company brands of reagents used in this study are listed in the Supplementary Table 3.

### 2.2. Human Nucleus Pulposus Specimens

Tissues used in this study were obtained from Sun Yat-sen Memorial Hospital of Sun Yat-sen University (Guangzhou, China). A total of three normal and six degenerative human NP specimens were obtained in this study. The degenerative nucleus pulposus tissues were collected from patients who underwent discectomy surgery due to disc herniation, while control specimens were obtained from patients who underwent surgery due to trauma without disc degeneration. After being collected, the tissues were fixed, embedded, and sectioned for use in experiments. Any extra tissues were put through a high-throughput tissue grinder for total RNA and protein extraction.

### 2.3. Puncture-Induced Rat IDD Model

Animal experiments were approved by the Institutional Research Ethical Committee of Sun Yat-sen University (No. SYSU-IACUC-2022-B0403). As previously described [32], briefly, a total of eighteen 12-week-old rats were housed in a vivarium with a 12-hour light/dark cycle. After being anesthetized, the rats were placed on a board, and 21 G needle punctures were performed in two adjacent caudal intervertebral discs at a depth of approximately 5 mm. After 1 minute, the needles were rotated 180 degrees and located for another 1 minute and then pulled out. The rats were divided into three groups: the control group, the punctured group, and the punctured with irisin treatment group. Irisin (100 *μ*g/kg) was injected intraperitoneally once a week in the punctured with irisin treatment group, and PBS (100 *μ*g/kg) solution was injected intraperitoneally once a week in the control group and the punctured group. After 4 weeks, a micro-MRI was performed and the degeneration of disc was assessed by the Pfirrmann grading system.

### 2.4. Human Nucleus Pulposus Cell Cultures

Human nucleus pulposus cells were purchased from ScienCell. Cells were incubated in human NP cell medium (ScienCell) with fetal bovine serum and antibiotics. When the confluence reached about 70%-90%, the cells were trypsinized, counted, and passaged. Cells from passages 4 to 7 were cultured in 6-well plates with about 8 ml medium. When they adhered, the medium was changed and the following treatments were performed.

### 2.5. Real-Time qPCR

Total RNA was extracted from cells or tissues with the Trizol reagent. Using RNA-iso Plus reagent and PrimeScript RT Master Mix (TaKaRa, Dalian, China), the RNA was converted to cDNA, and then, qPCR was performed. Firstly, the mix was heated to denaturation at 95°C for 5 minutes, and then, 40 cycles were performed (95°C for 10 seconds, 60°C for 20 seconds, and 72°C for 20 seconds). Finally, the dissolution curve was measured and the mRNA expression levels of different genes were calculated and analyzed, referring to the level of the *GAPDH* gene. The primers' sequences used in this study are listed in Supplementary Table 1.

### 2.6. Immunohistochemistry

Human and animal tissues were obtained and fixed with 4% paraformaldehyde for 24 to 48 hours and then decalcified and embedded in paraffin. The tissues were cut into sections about 5 *μ*m thickness. After being treated with 0.1% Triton X-100 solution, the sections were incubated with 3% peroxidase and then washed with PBS. For blocking, the bovine serum albumin was then used at room temperature for half an hour. After being incubated with antibodies (anti-COL2A1, anti-ACAN, anti-ADAMTS4, anti-TNF*α*, anti-MMP9, and anti-YAP) overnight, the Histostain Plus Kit (ZSGB-Bio, Beijing, China) was used for IHC analysis. Finally, an Olympus BX63 microscope was used for photographing. The dilutions of antibodies used in the study are listed in Supplementary Table 2.

### 2.7. Western Blot Analysis

Proteins from tissues and adherent cells were extracted using RIPA lysis buffer. Then, samples were added to SDS-PAGE to separate different proteins and transferred to PVDF or NC membranes. Subsequently, 5% nonfat dry milk was used for blocking, and the designated antibodies were added at 4°C overnight. Next day, the membranes were washed with PBS, and the secondary antibodies were added for 1 hour at 37°C. Finally, they were visualized using an ECL kit (Millipore), and the bands were quantified using ImageJ software. The dilutions of antibodies used in the study are listed in Supplementary Table 2.

### 2.8. Immunofluorescence

For cell immunofluorescence, the glasses were put in 24-well plates, and nucleus pulposus cells were seeded for about 24 h under different treatments. Next, the cells were fixed, blocked, and incubated with antibodies overnight. The next day, secondary antibodies were used for additional incubation for 1 h at 37°C, and then, they were labelled with DAPI. For tissue immunofluorescence, the tissues were decalcified, embedded, and treated with 0.1% Triton X-100 solution and bovine serum albumin. Then, they were incubated with antibodies at 4°C overnight. The next day, the tissues were incubated with secondary antibodies for 1 h, and then, they were labelled with DAPI. Finally, an Olympus BX63 microscope was used for photographing. The dilutions of antibodies used in the study are listed in Supplementary Table 2.

### 2.9. HE Staining and Safranin-O-Fast Green Staining

After being fixed, decalcified, dehydrated, and embedded, the rat intervertebral disc tissues were treated with hematoxylin for 2 min, followed by eosin for 3 min at 37°C for HE staining. As for Safranin-O-Fast Green staining, the sections were stained with Safranin-O for 15 min and then with Fast Green solution for 2 min. At the indicated time, the sections were dried and sealed with neutral resin. All sections were photographed using an Olympus BX63 microscope.

### 2.10. Cell Viability Assay

The NPCs were plated in 96-well plates at a density of approximately 1.0 × 10^4^ cells/ml. When the cells adhered, irisin was added at different concentrations (0, 25, 50, 100, 200, and 400 ng/ml) for 24 and 48 hours. At the indicated time, the CCK-8 reagent was added according to the manufacturer's instructions. Finally, absorbance at 450 nm was measured by using the microplate reader.

### 2.11. Transcriptome Sequencing and Bioinformatics Analysis

Genome-wide transcriptional sequencing was performed on both TNF-*α*-treated groups and TNF-*α* with irisin-treated groups to identify the differential expression levels of RNA. Sequencing was performed using Oebiotech (Shanghai Co., Ltd.). Using the DESeq2 R package, differential expression analysis was performed between the two groups, and the expression levels of genes were screened out when the fold change was larger than 2. Next, Gene Ontology (GO) enrichment analysis, including biological processes, cellular components, and molecular function, was performed on the following website (https://david.ncifcrf.gov). Finally, Kyoto Encyclopedia of Genes and Genomes (KEGG) pathways enrichment analysis was performed by Oebiotech (Shanghai Co., Ltd.).

### 2.12. Cell Transduction

*CTGF*-shRNA and control plasmid vectors were constructed by GeneChem (Shanghai, China). In brief, when the human NPCs were cultured and reached 70-90% confluence, the *CTGF*-shRNA plasmids were added to the medium with the Lipofectamine 3000 reagent. The concentration of DNA was about 2500 ng per well of the 6-well plate. After 8 hours, the medium was replaced, and regular culture was performed for the following experiments.

### 2.13. Statistical Analysis

The quantitative data shown in this study are presented as the mean ± standard deviation. Two-tailed Student's *t*-tests were conducted for comparisons between two groups, and the whole statistical analysis was performed using the SPSS 20.0 statistical software package (SPSS, Inc., Chicago, IL, USA). Results were regarded as statistically significant with a *P* value of less than 0.05.

## 3. Results

### 3.1. Decreased Expression of FNDC5 in Degenerative Human NP Tissue

**Figure 1:**
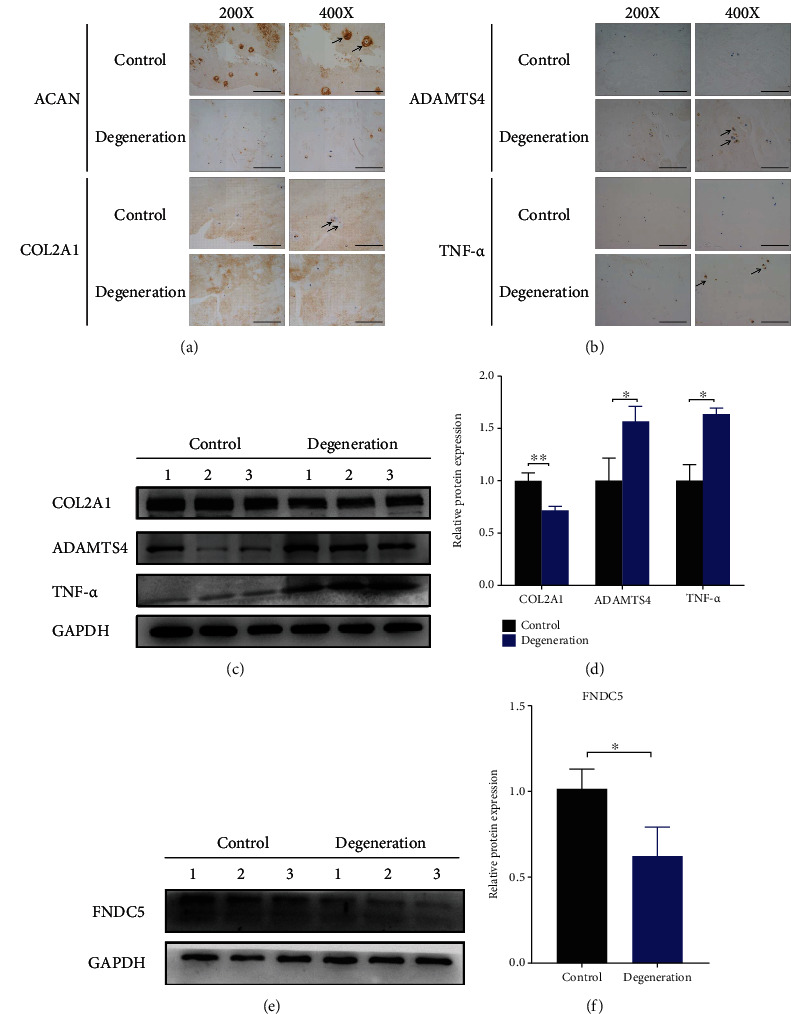
Decreased expression of FNDC5 in degenerative human NP tissue. (a, b) IHC staining assay of ACAN, COL2A1, ADAMTS4, and TNF-*α* in control and degenerative human nucleus pulposus tissues. (c) The protein expression levels of COL2A1, ADAMTS4, and TNF-*α* were detected using western blotting in control and degenerative human NP tissues. (d) The quantitative analysis of the protein bands in (c) using ImageJ software. (e) The protein expression levels of FNDC5 were detected by western blot in the control and degenerative groups. (f) The quantitative analysis of the protein bands in (e) using ImageJ software. The scale bar of images in (a) and (b) was shown (magnification: ×200, scale bar: 100 *μ*m; magnification: ×400, scale bar: 50 *μ*m). ^∗^*P* < 0.05, ^∗∗^*P* < 0.01.

Firstly, both the dysregulated ECM metabolism and inflammation levels were validated between control and degenerative human NP tissues. As shown in Figures 1(a) and 1(b), compared with the control group, the results from IHC revealed that the expression levels of anabolism markers (COL2A1 and ACAN) were suppressed, while the expression levels of ADAMTS4 and TNF-*α* was elevated, respectively (Figures 1(a) and 1(b)). Next, total proteins were extracted from tissues. As expected, using western blot, a decreased level of COL2A1 and increased levels of ADAMTS4 and TNF-*α* were found in degenerative groups (Figures 1(c) and 1(d)). To elucidate the change of FNDC5 in the progression of IDD, the expression level of the FNDC5 protein was also detected. Less expression of FNDC5 was found in degenerative NP tissues (Figures 1(e) and 1(f)). These results indicated that unbalanced ECM metabolism, increased TNF-*α*, and decreased FNDC5 expression levels existed in degenerated NP tissues.

### 3.2. Irisin Ameliorates the Progression of IDD in a Puncture-Induced Rat Model In Vivo

**Figure 2:**
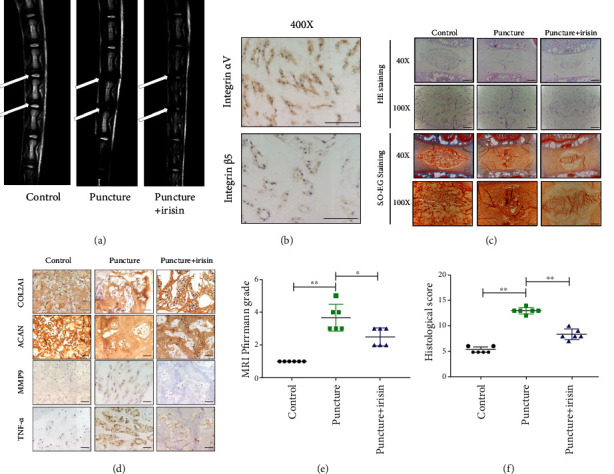
Irisin ameliorates the progression of IDD in a puncture-induced rat model in vivo. (a) The T2 phase images of micro-MRI scan of the caudal intervertebral disc in different groups: the white arrows indicated the control and affected discs. (b) The expression of the integrin *α*V*β*5 receptor in the intervertebral disc tissues (magnification: ×400, scale bar: 50 *μ*m). (c) HE staining of the affected intervertebral disc in different groups. Images (magnification: ×40, scale bar: 500 *μ*m; magnification: ×100, scale bar: 200 *μ*m). (d) IHC staining assay of COL2A1, ACAN, MMP9, and TNF-*α* in different groups. (e) The MRI Pfirrmann grade analysis of the intervertebral disc in different groups. (f) The histological score of the intervertebral disc in different groups. Images (magnification: ×400, scale bar: 50 *μ*m). ^∗^*P* < 0.05, ^∗∗^*P* < 0.01.

To assess the roles of irisin on the progression of IDD, puncture-induced rat models were established. The rats were injected with PBS or irisin reagent at a concentration of 100 *μg*/kg once a week. After 4 weeks, the rats were anesthetized, underwent micro-MRI examinations, and then were sacrificed. The results from the MRI T2 phase showed that the water content of NP tissues in the punctured group was significantly decreased compared to that in the controls, whereas irisin treatment reserved this change partly (Figure 2(a)). Firstly, the expressions of irisin receptors integrin *α*V and integrin *β*5 were detected in the intervertebral disc tissues (Figure 2(b)). Moreover, histological morphology was assessed by HE staining and Safranin-O-Fast Green staining. The results revealed a disordered disc structure, accompanied by the loss of a matrix and an unclear boundary between the annulus fibrosus and NP tissues in the punctured group. Meanwhile, irisin treatment partially restored the matrix synthesis and maintained the structure of the intervertebral disc (Figure 2(c)). In addition, IHC was performed to evaluate the metabolism of ECM. Compared with the controls, lower expression levels of synthesis markers (COL2A1, ACAN) and higher expression levels of matrix-degrading enzyme (MMP9) and TNF-*α* were found in the punctured group; those changes were also reversed with irisin treatment (Figure 2(d)). The Pfirrmann grades and the histological score of the punctured group were higher than those of the control group, but irisin treatment decreased the scores, respectively (Figures 2(e) and 2(f)). These results revealed that irisin treatment could ameliorate the unbalanced metabolism of ECM and alleviate the development of IDD *in vivo*.

### 3.3. Irisin Treatment Restored the Metabolism of the ECM in TNF-*α*-Induced NPCs

**Figure 3:**
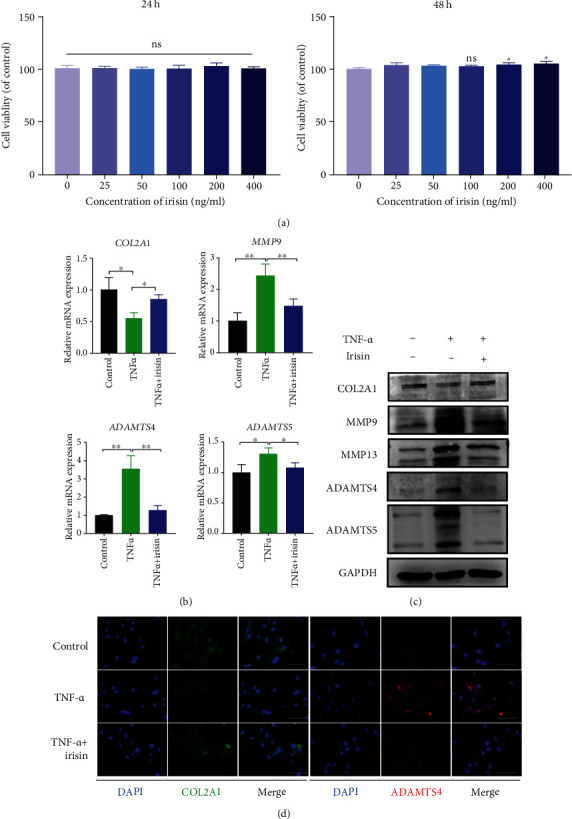
Irisin treatment restored the metabolism of the ECM in TNF-*α*-induced NPCs. (a) CCK-8 assay was performed to detect the effects of irisin on the cells' viability at various concentrations (0, 25, 50, 100, 200, and 400 ng/ml) at 24 and 48 hours. (b) The relative expression levels of different genes (COL2A1, MMP9, ADAMTS4, and ADAMTS5) were detected using qPCR in different groups. (c) The protein expression levels of COL2A1, MMP9, MMP13, ADAMTS4, and ADAMTS5 were detected by western blot in different groups. (d) The expression levels of COL2A1 and ADAMTS4 were detected by immunofluorescence in different groups. DAPI was used to stain for the nuclei. Images (magnification: ×400, scale bar: 50 *μ*m). In (b)–(d), irisin was used at the concentration of 100 ng/ml and TNF-*α* was used at the concentration of 10 ng/ml. ^∗^*P* < 0.05, ^∗∗^*P* < 0.01.

To determine the effects of irisin on NPCs, a CCK-8 assay was first performed, in which irisin treatment was found not to affect cell viability at a concentration below 100 ng/ml at both 24 and 48 hours (Figure 3(a)). Thus, irisin was used for following experiments at a concentration of 100 ng/ml. Subsequently, TNF-*α* was used to create an inflammatory microenvironment at a concentration of 10 ng/ml, as previous studies had reported [8, 9]. The anabolic gene marker *COL2A1* was found to be downregulated upon TNF-*α* treatment, while the catabolic gene markers (*MMP9*, *ADAMTS4*, and *ADAMTS5*) were upregulated. These changes were partly reversed with irisin treatment (Figure 3(b)). Similarly, the results from western blot were consistent with the mRNA change (Figure 3(c)). The protein expression level was further investigated by immunofluorescence, and it was found that irisin treatment had an antagonistic effect against TNF-*α*-induced ECM metabolism disorder (Figure 3(d)). Together, these data suggested that irisin inhibited catabolism and promoted anabolism of ECM in TNF-*α*-stimulated NPCs.

### 3.4. Irisin Treatment Affected the Activation of the Hippo Signaling Pathway

**Figure 4:**
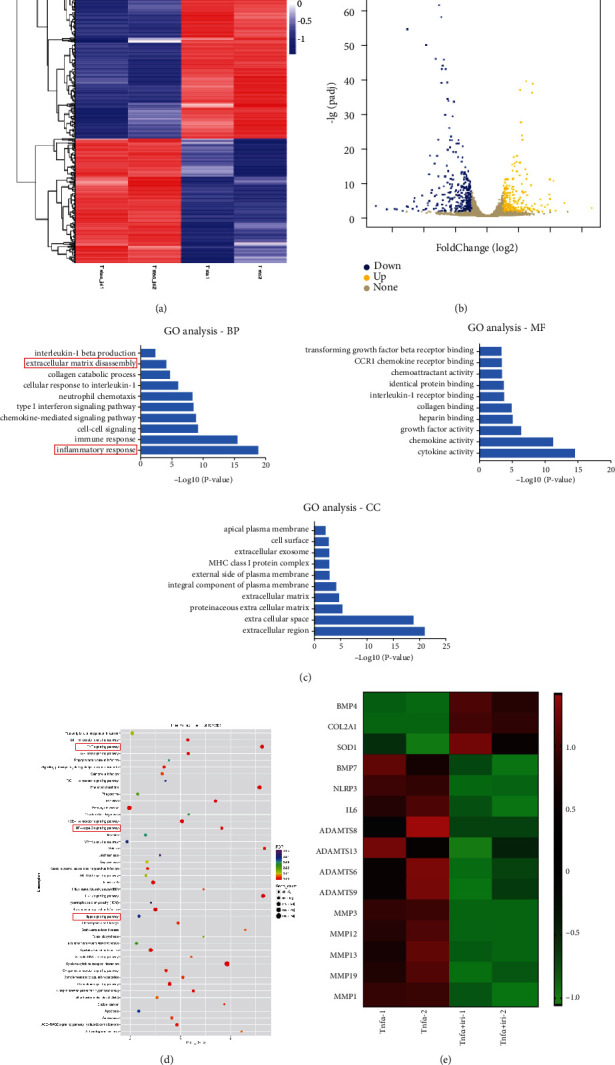
Irisin treatment affected the activation of the Hippo signaling pathway. (a) The heatmap of differentially expressed genes (DEGs), which were detected by RNA-seq in human nucleus pulposus cells between TNF-*α*-treated groups and TNF-*α* plus irisin-treated groups. (b) The volcano map of gene distribution (upregulation, downregulation, and none). (c) GO analysis terms with the most significant *P* values were determined, including three analyses (BP: biological process, CC: cellular component, and MF: molecular function). (d) KEGG pathway enrichment analysis showed the affected signaling pathways with *P* value less than 0.05. (e) The heatmap of genes' expression levels, including anabolic marker (*COL2A1*), catabolic markers (*MMPs* and *ADAMTSs*), and inflammation factors (*IL-6* and *NLRP3*). In this figure, irisin was used at the concentration of 100 ng/ml and TNF-*α* was used at the concentration of 10 ng/ml.

To further explore the potential mechanism of irisin treatment on the progression of IDD, RNA-seq was performed on human NPCs from both TNF-*α*-treated groups and TNF-*α* plus irisin-treated groups. As shown in Figure 4(a), a total of 531 genes were identified as differentially expressed (fold change > 2 and adjusted *q* value < 0.05). Among these, 212 genes were upregulated and 319 genes were downregulated with irisin treatment (Figures 4(a) and 4(b)). Next, Gene Ontology analysis was performed and the results revealed that inflammatory response and extracellular matrix disassembly were the affected terms under irisin treatment (Figure 4(c)). Kyoto Encyclopedia of Genes and Genomes analysis revealed that the TNF, NF-*κ*B, and Hippo signaling pathways were modulated under irisin treatment (Figure 4(d)). Notably, the expression levels of ADAMTSs (*ADAMTS6*, *ADAMTS8*, *ADAMTS9*, and *ADAMTS13*), MMPs (*MMP1*, *MMP3*, *MMP13*, *MMP16*, and *MMP19*), and inflammatory factors (*IL-6* and *NLRP3*) were found to be significantly downregulated, while the matrix anabolic gene *COL2A1* was markedly upregulated in the irisin-treated group (Figure 4(e)). Collectively, these results indicated that irisin treatment was involved in different biological processes in TNF-*α*-induced NPCs as well as regulating the activation of the Hippo signaling pathway.

### 3.5. LATS and YAP Mediated the Effects of Irisin on the ECM Metabolism in Human NPCs

**Figure 5:**
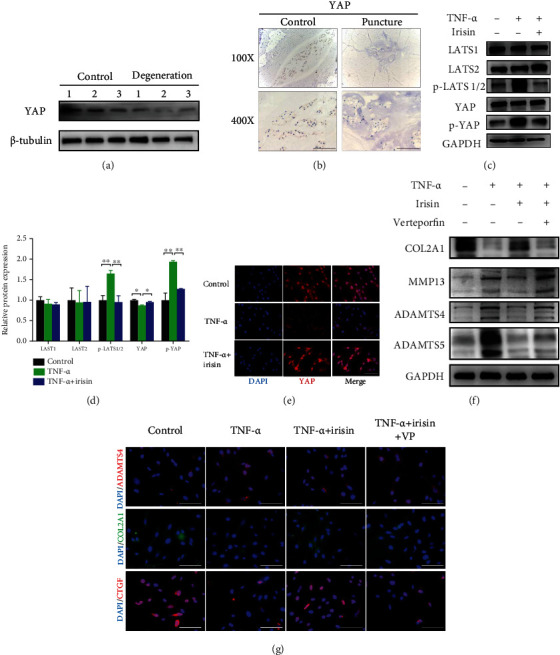
LATS and YAP mediated the effects of irisin on the ECM metabolism in human NPCs. (a) The expression levels of YAP protein were detected by western blot between control and degenerative groups. (b) IHC staining was performed to detect the YAP expression level between control and puncture groups in rats' intervertebral disc. Images (magnification: ×400, scale bar: 50 *μ*m). (c) Protein expression levels of the Hippo signaling pathway (LATS1, LATS2, p-LATS1/2, YAP, and p-YAP) were detected by western blot in different groups. (d) Quantitative analysis of the bands in (c) using ImageJ software. (e) The localization of YAP was assessed via immunofluorescence in different groups. Images (magnification: ×400, scale bar: 50 *μ*m). (f) The protein expression levels of ECM metabolism markers (COL2A1, MMP13, ADAMTS4, and ADAMTS5) were assessed by western blot in human NPCs. (g) The expression levels of ADAMTS4, COL2A1, and CTGF were detected by immunofluorescence in human NPCs. Images (magnification: ×400, scale bar: 50 *μ*m). In this figure, irisin was used at the concentration of 100 ng/ml, TNF-*α* was used at the concentration of 10 ng/ml, and verteporfin was used at the concentration of 5 *μ*mol/l. ^∗^*P* < 0.05, ^∗∗^*P* < 0.01.

To further explore the function of the Hippo signaling pathway in the progression of IDD, the expression levels of key factors of the Hippo signaling pathway were investigated, and the expression level of YAP was found to be downregulated in human degenerative NP tissue (Figure 5(a)). Similar results were detected in punctured-induced rat intervertebral disc tissues by IHC (Figure 5(b)). *In vitro*, it was examined whether the phosphorylation levels of LATS and YAP were affected. The phosphorylation levels of LATS and YAP were found to be increased in TNF-*α*-induced NPCs, while irisin treatment significantly reversed these changes (Figures 5(c) and 5(d)). Moreover, the results of immunofluorescence revealed that less YAP was imported into the nucleus after TNF-*α* stimulation, while irisin treatment clearly promoted YAP to enter the nucleus in NPCs (Figure 5(e)). To further explore whether the Hippo signaling pathway was involved in irisin-treated NPCs, verteporfin, a well-established YAP inhibitor, was applied. The levels of COL2A1 were found to be upregulated, while the expression levels of matrix-degrading enzymes (MMP13, ADAMTS4, and ADAMTS5) were downregulated with irisin treatment in TNF-*α*-induced NPCs, which was abolished upon verteporfin treatment (Figure 5(f)). As expected, similar results were found by using immunofluorescence. Moreover, the expression level of connective tissue growth factor (CTGF), as the target molecule of YAP, was found to be significantly affected with irisin treatment (Figure 5(g)). Together, these data showed that LATS and YAP were involved in irisin-mediated ECM metabolism in NPCs.

### 3.6. CTGF Protein Mediated the Protective Effect of Irisin on the Regulation of ECM's Metabolism in Human NPCs

**Figure 6:**
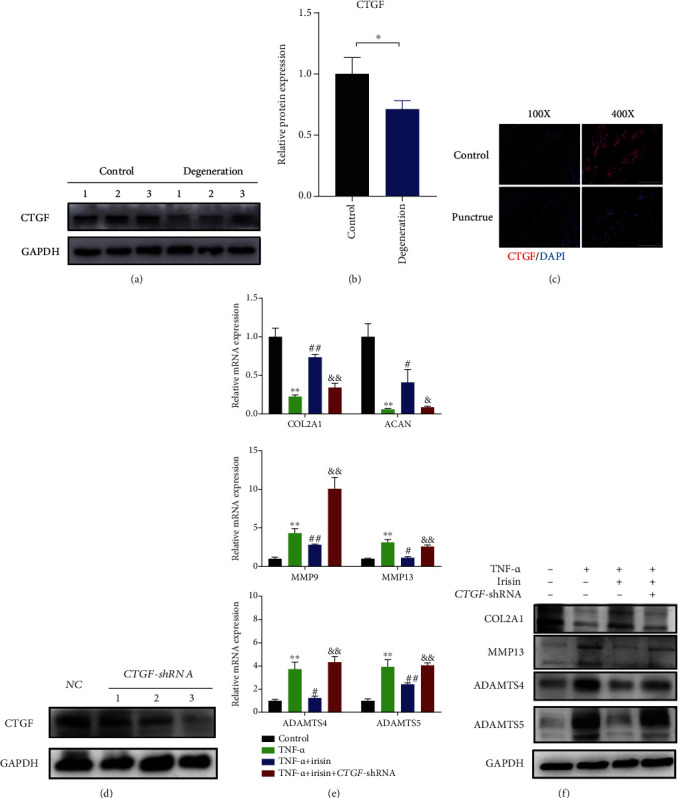
CTGF protein mediated the protective effect of irisin on the regulation of ECM metabolism in human NPCs. (a) The expression levels of CTGF protein were detected between control and degenerative groups. (b) Quantitative analyses of the bands in (a) using ImageJ software. (c) Immunofluorescence analysis was performed between control and puncture groups in rats' intervertebral disc. Images (magnification: ×400, scale bar: 50 *μ*m). (d) The transfection efficiency verification of CTGF using western blot. (e) The mRNA expression levels of anabolic markers (*COL2A1* and *ACAN*) and catabolic markers (*MMP9*, *MMP13*, *ADAMTS4*, and *ADAMTS5*) were detected by qPCR. (f) The protein levels of COL2A1, MMP9, ADAMTS4, and ADAMTS5 were assessed by western blot. In this figure, irisin was used at the concentration of 100 ng/ml and TNF-*α* was used at the concentration of 10 ng/ml. ^∗^*P* < 0.05, ^∗∗^*P* < 0.01 compared with the control group. ^#^*P* < 0.05, ^##^*P* < 0.01 compared with the TNF-*α* group. ^&^*P* < 0.05, ^&&^*P* < 0.01 compared with the TNF-*α*+irisin group.

We then detected the expression levels of target genes of the Hippo signaling pathway, such as *CTGF* and *CYR61*, and found that the mRNA expression levels of *CTGF* were lower than those in the normal group (Supplementary Figure 1). To further assess the effects of CTGF on the progression of IDD, the expression levels of CTGF were examined in both human control and degenerative NP tissues. The protein expression level of CTGF was lower in degenerative tissues than in the controls (Figures 6(a) and 6(b)). Similar results were found in a rat's intervertebral disc tissue by immunofluorescence (Figure 6(c)). Next, *CTGF* knockdown in human NPCs was performed by transfecting plasmid, and verification of transfection efficiency was shown using western blot (Figure 6(d)). Subsequently, the irisin reagent was added, and irisin treatment was found to ameliorate the unbalanced metabolism of ECM, while these effects were antagonized in the *CTGF-*shRNA group in both mRNA and protein levels (Figures 6(e) and 6(f)). Collectively, the data showed that the CTGF protein participated in the regulative effects of irisin on NPCs, suggesting that irisin could ameliorate an unbalanced ECM metabolism via LATS/YAP/CTGF signaling *in vitro*.

## 4. Discussion

Intervertebral disc degeneration (IDD), regarded as one of the primary causes of low back pain (LBP), is accompanied by unbalanced ECM metabolism, tissue dehydration, fissures in the annulus fibrosus, and destruction of endplate cartilage [3, 39, 40]. Currently, it remains a significant challenge to delay or even reverse the progression of IDD due to limited available treatments. Irisin, also known as FNDC5, is considered to show immense potential for application in the fields of bone and cartilage degenerative diseases. Irisin treatment was discovered to improve bone metabolism and thus prevented the osteoporosis [22, 41]. Regarding the regulation of cartilage degenerative diseases, irisin was found to display chondroprotective effects on the development of osteoarthritis and played a powerful role in the regulation of ECM metabolic disorder [25, 26]. Nevertheless, the effects of irisin on NPCs are not well known yet.

**Figure 7:**
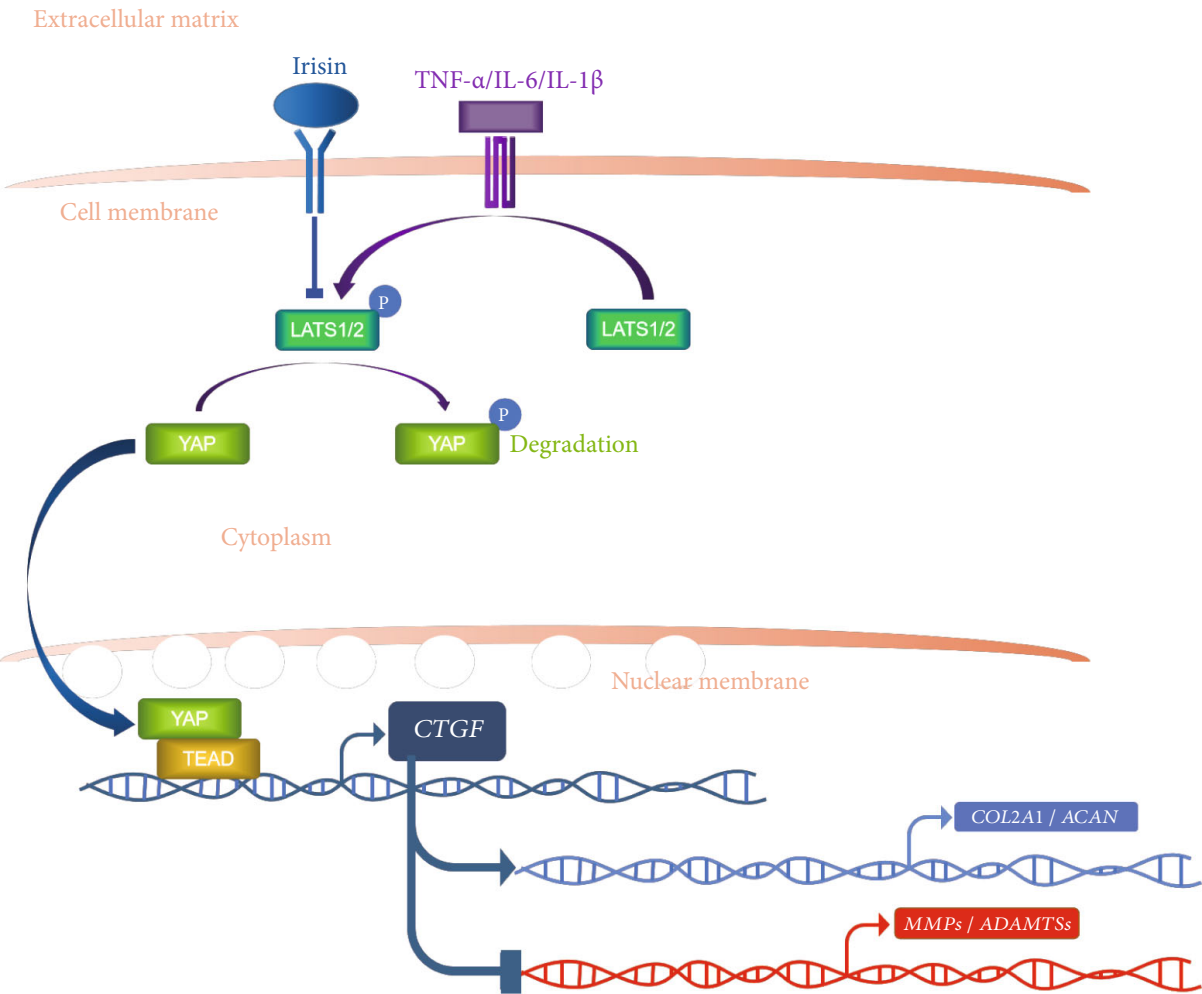
Schematic diagram of irisin's effects on human NPCs. Irisin inhibited the phosphorylation of LATS and YAP proteins induced by TNF-*α*, promoting nucleus translocation of YAP and upregulating the expression level of CTGF, thus promoting the expression levels of matrix synthesis markers (*COL2A1* and *ACAN*) and inhibiting the levels of matrix-degrading enzymes (*MMPs* and *ADAMTSs*), finally reversing the unbalanced metabolism of ECM in human NPCs.

In this study, irisin was found to play a protective role on the ECM metabolism of NPCs, and it delayed the progression of IDD in rat models. In mechanism, it was demonstrated that irisin treatment regulated LATS/YAP/CTGF signaling, inhibiting the phosphorylation of LATS and YAP and promoting the expression level of CTGF protein. Finally, the levels of synthetic markers (*COL2A1* and *ACAN*) were upregulated and the expression levels of matrix-degrading enzymes (*MMPs* and *ADAMTSs*) were downregulated, and the disordered metabolism of ECM in NPCs was reversed (Figure 7). The research in this study evidenced the positive effects of irisin on NPCs and its possible mechanism, suggesting the possible application of irisin in the field of IDD.

Ample studies have shown that the Hippo signaling pathway is related closely to mechanical stress and is involved in bone and cartilage degenerative diseases [42, 43]. According to the available evidence, the YAP protein has been reported to promote the anabolic metabolism of ECM in NPCs [31, 32]. CTGF, as the target molecule of the Hippo signaling pathway, has been reported to be involved in the ECM metabolism of nucleus pulposus cells [31, 32]. Nevertheless, the mechanisms governing LATS/YAP/CTGF in the regulation of NPCs have remained elusive. The results of this study revealed that irisin treatment inhibited the phosphorylation levels of LATS and the YAP protein and effectively promoted the level of the CTGF protein, suggesting that irisin prevented the deterioration of ECM metabolism and ameliorated the progression of IDD.

According to available studies, 80% of the population has suffered from LBP in their lifetime, which may be a serious burden due to the recurring symptoms [44]. IDD is currently regarded as the main etiology of LBP. However, there is still a lack of effective treatments for delaying or even reversing IDD [45]. In the current study, a new potential treatment was proposed. Irisin was proven to have obvious effects on regulating ECM metabolism in NPCs, suggesting that it may be a potential therapeutic drug for the treatment of IDD. More *in vivo* studies for the applications of irisin will be needed to explore its potential in the future.

## 5. Conclusion

Taken together, this study demonstrated the protective effects of irisin on the development of IDD. Irisin treatment could significantly reverse the unbalanced metabolism of ECM in NPCs, thus delaying the progression of IDD. Mechanistically, it was identified that irisin inhibited the phosphorylation of LATS and YAP proteins, promoting the YAP protein into the nucleus and upregulating the expression level of CTGF, thus promoting the expression levels of matrix synthesis markers (COL2A1 and ACAN) and inhibiting the levels of matrix-degrading enzymes (MMPs and ADAMTSs). This study provided more theoretical basis for the application of irisin in the field of IDD.

## Data Availability

The data used to support the findings of this study are available from the corresponding authors upon request.

## Supplementary Materials

Supplementary 1 Figure S1: the expression levels of genes (*CTGF* and *CYR61*) between control and degenerative groups were detected by qPCR. ^∗∗^*P* < 0.01 compared with the control group. ns: no statistical difference.

Supplementary 2 Table S1: primers' sequences for real-time qPCR. Table S2: dilutions of antibodies used in this study. Table S3: catalog numbers and company brands of reagents used in this study.
