# Supplementary material for: Irisin Ameliorates Intervertebral Disc Degeneration by Activating LATS/YAP/CTGF Signaling
Source: Oxid Med Cell Longev. 2022 Jul 20;2022:9684062. doi: 10.1155/2022/9684062 (PMC9338732; doi:10.1155/2022/9684062)
Supplement: Supplementary 1 — Figure S1: the expression levels of genes (CTGF and CYR61) between control and degenerative groups were detected by qPCR. ∗∗P < 0.01 compared with the control group. ns: no statistical difference. [file 9684062.f1.docx]

**Supplement Figures and figure legends**

**Irisin ameliorates the progression of intervertebral disc degeneration via LATS/YAP/CTGF signaling.**

**Fig. S1**

**
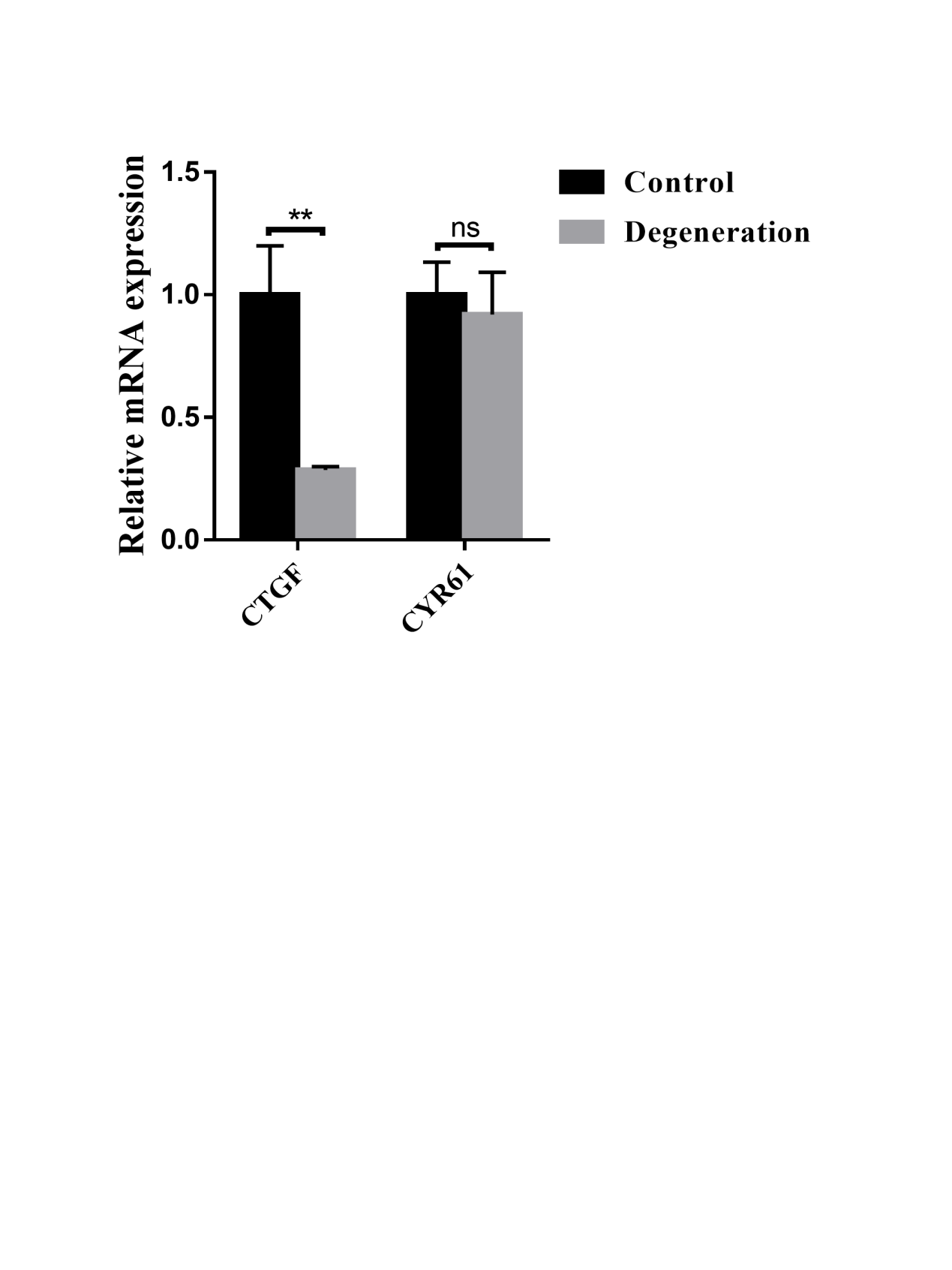
**

**Fig. S1** The expression levels of genes (*CTGF*, and *CYR61*) between control and degenerative groups were detected by qPCR. ^**^*P* < 0.01 compared with the control group. ns: no statistical difference.
