# Supplementary material for: Irisin Ameliorates Intervertebral Disc Degeneration by Activating LATS/YAP/CTGF Signaling
Source: Oxid Med Cell Longev. 2022 Jul 20;2022:9684062. doi: 10.1155/2022/9684062 (PMC9338732; doi:10.1155/2022/9684062)
Supplement: Supplementary 2 — Table S1: primers' sequences for real-time qPCR. Table S2: dilutions of antibodies used in this study. Table S3: catalog numbers and company brands of reagents used in this study. [file 9684062.f2.docx]

**Table S1**. Primers’ sequences for Real-time qPCR.

| Gene primer names | Primer sequence(5’-3’) |
| --- | --- |
| *GAPDH*-F  *GAPDH*-R  *COL2A1*-F  *COL2A1*-R  *MMP9*-F  *MMP9*-R  *ADAMTS4*-F  *ADAMTS4*-R  *ADAMTS5*-F  *ADAMTS5*-R  *ACAN*-F  *ACAN*-R  *MMP13*-F  *MMP13*-R | AGAAAAACCTGCCAAATATGATGAC  TGGGTGTCGCTGTTGAAGTC  GGCAATAGCAGGTTCACGTACA  GAACATCGACCAACTCTACTCCG  TGTACCGCTATGGTTACACTCG  GGCAGGGACAGTTGCTTCT  GAGGAGGAGATCGTGTTTCCA  CCAGCTCTAGTAGCAGCGTC  GAACATCGACCAACTCTACTCCG  CAATGCCCACCGAACCATCT  ACTCTGGGTTTTCGTGACTCT  ACACTCAGCGAGTTGTCATGG  ACTGAGAGGCTCCGAGAAATG  GAACCCCGCATCTTGGCTT |

**Table S2**. Dilutions of antibodies used in this study.

| Antibodies | Dilution |
| --- | --- |
| GAPDH  β-tubulin  COL2A1  ACAN  ADAMTS4  TNF-α  FNDC5  MMP9  ADAMTS5  MMP13  YAP  p-YAP  LATS1  LATS2  p-LATS1/2  CTGF | WB (1:3000)  WB (1:3000)  WB (1:1000); IHC/IF (1:100)  WB (1:1000); IHC (1:100)  WB (1:1000); IHC/IF (1:100)  WB (1:1000); IHC (1:100)  WB (1:1000)  WB (1:1000); IHC (1:100)  WB (1:250)  WB (1:1000)  WB (1:1000) ；IHC/IF (1:100)  WB (1:1000)  WB (1:1000)  WB (1:1000)  WB (1:1000)  WB (1:1000) ; IF (1:100) |

**Table S3**. Catalog numbers and company brands of reagents used in this study.

| Reagents | Company, Cat# |
| --- | --- |
| Anti-GAPDH  Anti-β-tubulin  Anti-COL2A1  Anti-ACAN  Anti-ADAMTS4  Anti-TNF-α  Anti-FNDC5  Anti-MMP9  Anti-ADAMTS5  Anti-MMP13  Anti-YAP  Anti-p-YAP  Anti-LATS1  Anti-LATS2  Anti-CTGF  TNF-alpha  Irisin  Verteporfin | Proteintech Group Inc., Cat# 60004-1-Ig  Proteintech Group Inc., Cat# 10094-1-AP  Abcam Inc. Cat# ab34712 and ab188570  Abcam Inc. Cat# ab3778  Abcam Inc. Cat# ab185722 and ab84792  Abcam Inc. Cat# ab1793  Bioss Inc. Cat# bs8786R  Cell Signaling Technology Inc. Cat# 13667  Abcam Inc. Cat# ab41037  Cell Signaling Technology Inc. Cat# 69926  Cell Signaling Technology Inc. Cat# 14074  Cell Signaling Technology Inc. Cat# 53749  Cell Signaling Technology Inc. Cat# 9153  Cell Signaling Technology Inc. Cat# 5888  Cell Signaling Technology Inc. Cat# 86641  R&D Systems. Cat# 210-TA-100/CF  Novoprotein. Cat# CM35  MedChemExpress. Cat# HY-B0146 |
